# Insulin-Like Growth Factor Binding Protein-2 Level Is Increased in Blood of Lung Cancer Patients and Associated with Poor Survival

**DOI:** 10.1371/journal.pone.0074973

**Published:** 2013-09-17

**Authors:** Chengcheng Guo, Haibo Lu, Wen Gao, Li Wang, Kaihua Lu, Shuhong Wu, Apar Pataer, Maosheng Huang, Randa El-Zein, Tongyu Lin, Jack A. Roth, Reza Mehran, Wayne Hofstetter, Stephen G. Swisher, Xifeng Wu, Bingliang Fang

**Affiliations:** 1 Department of Thoracic and Cardiovascular Surgery, the University of Texas MD Anderson Cancer Center, Houston, Texas, United States of America; 2 Department of Epidemiology, the University of Texas MD Anderson Cancer Center, Houston, Texas, United States of America; 3 Department of Neurosurgery/Neuro-Oncology, Sun Yat-sen University Cancer Center, Guangzhou, China; 4 Department of Medical Oncology, Sun Yat-sen University Cancer Center, Guangzhou, China; 5 The 8^th^ Department of Internal Medicine, the Third Affiliated Hospital of Harbin Medical University, Harbin, China; Wayne State University, United States of America

## Abstract

**Background:**

We recently showed that IGFBP2 is overexpressed in primary lung cancer tissues. This study aims to determine whether IGFBP2 is elevated in blood samples of lung cancer patients and whether its level is associated with clinical outcomes.

**Methodology/Principal Findings:**

Plasma IGFBP2 levels were determined blindly by enzyme-linked immunosorbent assay in 80 lung cancer patients and 80 case-matched healthy controls for comparison. We analyzed blood samples for IGFBP2 levels from an additional 84 patients with lung cancer and then tested for associations between blood IGFBP2 levels and clinical parameters in all 164 lung cancer patients. All statistical tests were two-sided and differences with p<0.05 were considered significant. The mean plasma concentration of IGFBP2 in lung cancer patients was significantly higher than that in healthy controls (388.12±261.00 ng/ml vs 219.30±172.84 ng/ml, p<0.001). IGFBP2 was increased in all types of lung cancer, including adenocarcinoma, squamous cell cancer, and small-cell cancer, regardless of patients’ age, sex, or smoking status. IGFBP2 levels were mildly but significantly associated with tumor size and were significantly higher in stage IV than stage I or III disease. A multivariate analysis showed that lung cancer patients whose blood IGFBP2 was higher than 160.9 ng/ml had a poor survival outcome, with a hazard ratio of 8.76 (95% CI 1.12-68.34, p=0.038 after adjustment for tumor size, pathology, and stage). The median survival time for patients with blood IGFBP2 >160.9 ng/ml is 15.1 months; whereas median survival time was 128.2 months for the patients whose blood IGFBP2 was ≤160.9 ng/ml (p =0.0002).

**Conclusions/Significance:**

Blood IGFBP2 is significantly increased in lung cancer patients. A high circulating level of IGFBP2 is significantly associated with poor survival, suggesting that blood IGFBP2 levels could be a prognostic biomarker for lung cancer.

## Introduction

Despite the advent of new anticancer agents, lung cancer remains the leading cause of cancer mortality in the United States and worldwide [1]. A recent study showed that early diagnosis by low-dose helical computed tomography (CT) screening could significantly reduce the death rate by 6.7% for lung cancer patients [2], demonstrating that early diagnosis is the key to improving patients’ survival. Nevertheless, early diagnosis for lung cancer is challenging because low-dose CT screening is highly costly and is likely not affordable for the general population [3]. Moreover, predictive biomarkers for treatment response and prognosis are needed in clinics to guide therapeutic decision on various options [4,5]. Biomarkers that identify patient subgroups with significant different prognosis may provide helpful information on treatment strategies for those subgroups, such as whether an aggressive adjuvant therapy is needed for the patients with poor prognosis. Unfortunately, very few serological biomarkers that can be used for early diagnosis or prognosis of lung cancer are clinically available; therefore, identification of such biomarkers is highly desirable.

Insulin-like growth factor (IGF) binding proteins (IGFBPs) are a family of proteins (six in humans) that bind and serve as carriers of IGFs, prolonging the IGF half-life in the circulation and modulating local IGF concentrations and activities [6]. These proteins share a conserved three-domain structure, consisting of two conserved cysteine-rich domains at N- and C-terminals that are required for IGF binding and a non-conserved central domain that separates them [7]. In humans, IGFBP3 is the most abundant and IGFBP2 is the second most abundant IGFBP in the circulation. Unlike IGFBP3, which induces antitumor activity in different types of cancers [8,9], IGFBP2 has been shown to promote tumorigenesis [10], cancer cell invasion [11], metastasis [12], cancer stem cell expansion [13], and tumor angiogenesis [12,14] in various types of cancers. Indeed, IGFBP2 is overexpressed in the tumor tissues of glioma [15], breast cancer [16,17], ovarian cancer [18], prostate cancer [19], colorectal cancer [20], gastric cancer [21], lung cancer [22], leukemia [23], and astrocytoma [24]. Moreover, elevated serum or plasma IGFBP2 levels have been observed in patients with glioma [25], prostate cancer [26,27], ovarian cancer [28], and colon cancer [29,30]. Increased expression of IGFBP2 is implicated in either a shorter overall survival time [28,29,31] or resistance to chemotherapy [23,32]. Together, those results suggest that IGFBP2 could be a common oncogenic protein for various types of cancers.

To identify possible biomarkers for lung cancer, we recently determined the differential expression of proteins in primary lung tumor tissues and normal lung tissues from the same patients by using a reverse-phase protein array assay. IGFBP2 is one of the proteins found to be significantly increased in primary tumor tissues of non-small-cell lung cancers, including adenocarcinoma and squamous cell cancer [22]. Because IGFBP2 is a secretory protein, its increased expression in tumor tissues may be reflected in its concentrations in blood. To determine if that is true, we analyzed the IGFBP2 levels in plasma of lung cancer patients and healthy case-matched controls. Our results showed that the blood concentration of IGFBP2 was significantly increased in lung cancer patients and that a high IGFBP2 concentration in the patients’ blood was associated with short overall survival.

## Results

### Clinical characteristics of lung cancer patients

Clinical information for the 164 lung cancer patients in our study is summarized in Table 1. The patients (71 men and 93 women) were 42–86 years old, with a median age of 52. Adenocarcinoma and squamous cell carcinoma accounted for 54% and 21% of histological diagnosis, respectively. There were 33 patients with stage I disease, 13 patients with stage II disease, 40 patients with stage III disease, and 67 patients with stage IV disease. There were 138 patients (84%) who had a history of tobacco use/smoking. Patients who received surgery, chemotherapy, radiotherapy, or multi-modality therapy were 61 (37.2%), 105 (64.02%), 46 (28.05%), and 78 (46.34%), respectively. Six patients (0.37%) did not receive any therapy. There were 62 patients (37.8%) had metastasis at diagnosis, and 37 patients (22.56%) had local recurrence during the follow-up. The mean follow-up time was 37.5 months. The survival data were available for 152 patients, among them 83 (54.61%) were still alive by the time of data analysis. The healthy controls samples were matched with age, sex, race and smoking status of the first 80 lung cancer cases.

**Table 1:** Patient characteristics and plasma IGFBP2 levels (mean±SD).

**Variable**	**Patient(N**)	**IGFBP2 ng/ml**	**P Value**
Sex			
Male	71	382.4±248.8	
Female	93	303.6±209.7	0.024
Age			
<52 years	77	300.3±183.3	
≥52 years	87	370.8±261.4	0.129
Smoking status			
Never	26	306.3±216.0	
Current & Ever	138	343.6±233.0	0.349
Tumor Histology			
Adenocarcinoma	87	334.8±227.4	
Squamous-cell carcinoma	34	313.5±251.7	
Non-small-cell carcinoma	16	310.7±143.1	
Small-cell carcinoma	24	389.7±222.3	≥0.099^**^
Unknown/missing data	3		
Disease Stage			
Stage I^*^	33	259.4±187.4	0.001^*^
Stage II	13	402.3±282.6	
Stage III #	40	324.3±245.3	0.029#
Stage IV^*^#	67	383.6±226.4	≥0.07^**^
Unknown/missing data	11		
Metastasis			
No	92	305.7±229.8	
Yes	62	381.1±227.0	0.047
Unknown/missing Data	10		

^*^ P value for stage I versus stage IV; # for stage III versus stage IV; ^**^ for other groups in the same category

### Plasma IGFBP2 levels in lung cancer patients and in matched controls

**Figure 1:**
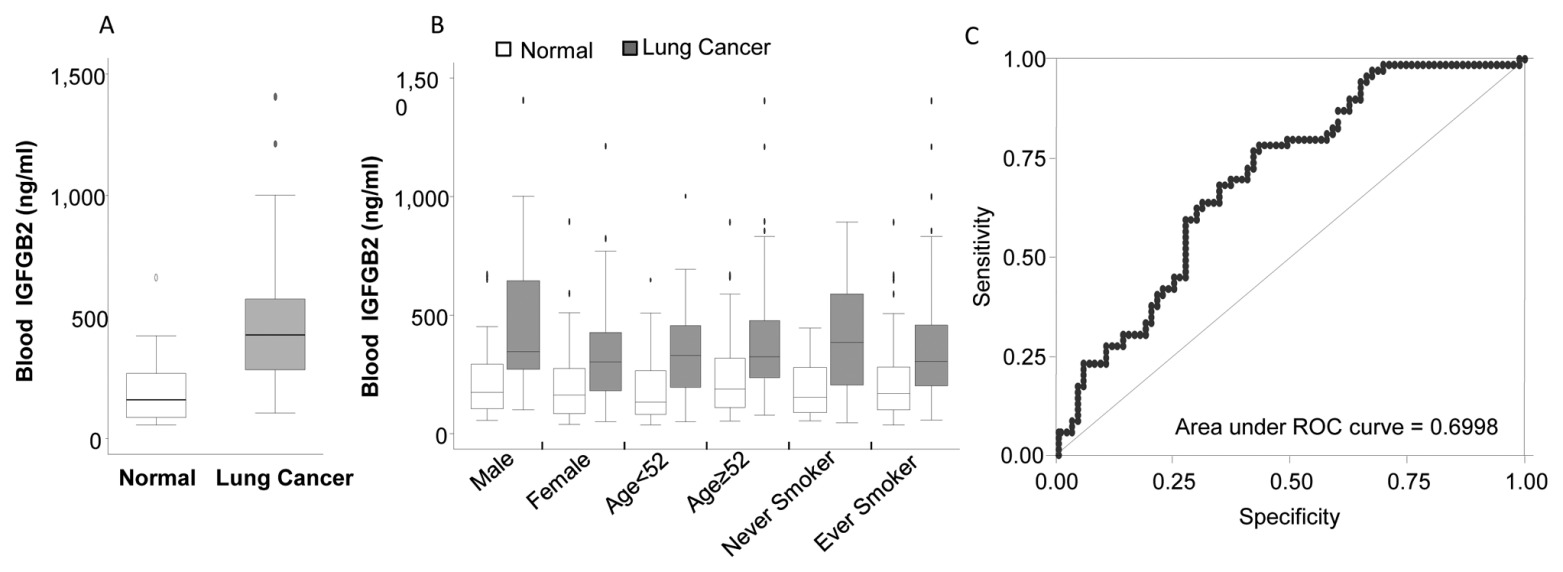
Plasma IGFBP2 levels in lung cancer patients (N=80) and the healthy controls (N=80). A) The box plots show mean (line inside the box) ± 1 SD (box) and ± 2 SD (the error bar). The difference between the mean values for the two groups is significant (*P*=2.4 x 10^-7^). B) Comparison of lung cancer patients and healthy controls on the basis of sex, age, and smoking status. In each group, the mean IGFBP2 values in lung cancer patients were significantly higher than those in healthy controls (*P*<0.01). C) The receiver operating characteristic curve (ROC) for diagnosis of lung cancer with plasma IGFBP2 values.

To test whether there is a difference in circulating IGFBP2 levels between lung cancer patients and healthy controls, we obtained plasma samples of 80 lung cancer patients and 80 case-matched healthy samples from the blood bank maintained at our institution’s department of epidemiology. The plasma IGFBP2 levels were determined blindly by ELISA without the investigator’s knowing any clinical information. IGFBP2 values for each coded sample were then analyzed statistically for association and correlation with clinical information. The results showed that the mean plasma concentration of IGFBP2 in lung cancer patients (388.12±261.00 ng/ml) was significantly higher than that in healthy controls (219.30±172.84 ng/ml, *P*=2.4 x 10^-7^) (Figure 1A). When the data were analyzed on the basis of sex, age, and smoking status, the IGFBP2 values remained significantly higher in lung cancer patients than in controls within each subgroup (Figure 1B). Within the healthy controls, the blood IGFBP2 level was significantly higher in those age 52 or older than those younger than 52 (mean 255.2 ng/ml vs 183.4 ng/ml, *P*=0.036). This result demonstrated that circulating IGFBP2 values were significantly higher in lung cancer patients, regardless of their age, sex, and smoking status.

### Specificity and sensitivity of using blood IGFBP2 for lung cancer diagnosis

We then determined the specificity and sensitivity of using plasma IGFBP2 levels for lung cancer diagnosis. The results showed that the receiver operating characteristic (ROC) plot had an area under curve of about 0.70 (Figure 1C). At a cutoff value of 160.9 ng/ml, the specificity and sensitivity of using IGFBP2 alone to differentiate the cancer and healthy groups were 76.92% and 75.61%, respectively. Fisher exact analysis showed that the difference between the two groups was significant (*P*= 6.01 x 10^-8^). The difference between the two groups remained significant when the specificity increased to 90% at a cutoff value of 437ng/ml, at which point sensitivity was about 30%. When the specificity increased to 95% (cutoff of 450ng/ml), however, the sensitivity was only about 13.8%, and the difference between the two groups was no longer significant (*P*=0.058). This result suggested that blood IGFBP2 values alone might be not sufficient for diagnosis of lung cancer because the optimal overall sensitivity and specificity values were about 70%. Nevertheless, IGFBP2 might be significantly increased in a subset of lung cancer patients.

### Circulating IGFBP2 in subgroups of lung cancer patients

**Figure 2:**
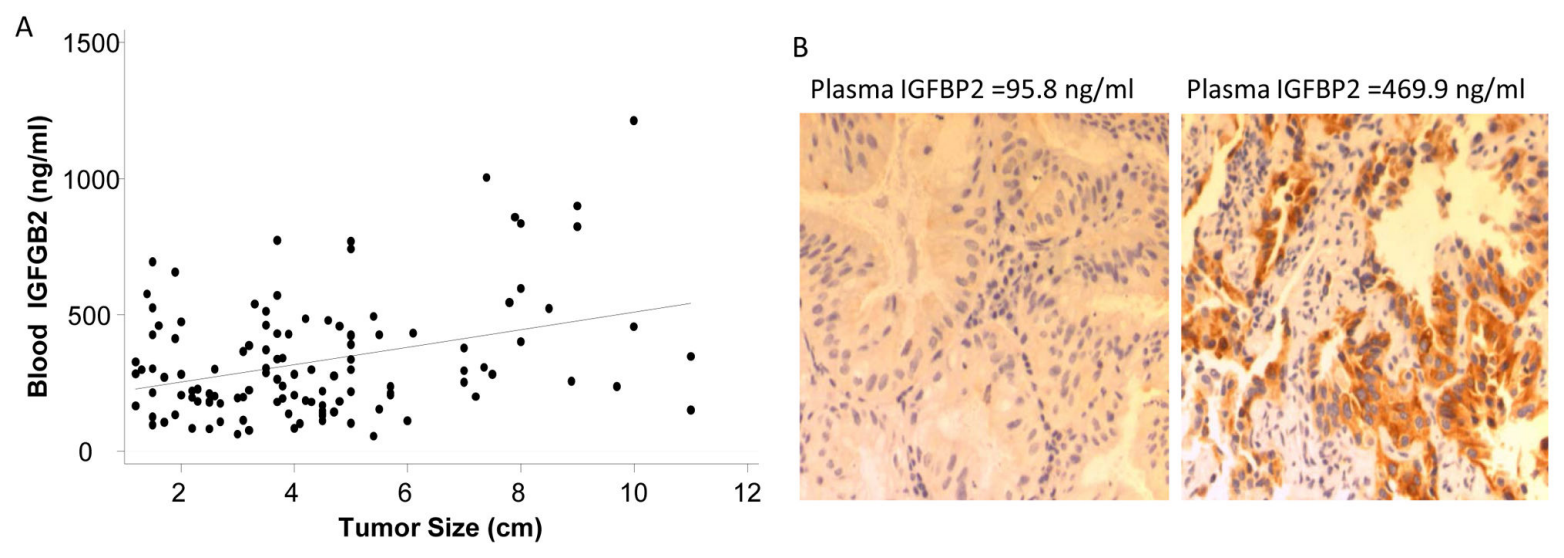
Association of blood IGFBP2 values with tumor sizes or IGFBP2 expression in primary tumors. A) Correlation between blood IGFBP2 values and tumor size (R=0.21, *P*=0.023). B) IGFBP2 expression in primary tumors of patients with low or high plasma IGFBP2. The levels of IGFBP2 in plasma were shown on the top of the panel.

In order to determine which subgroup of lung patients might have increased blood IGFBP2 levels, we determined IGFBP2 levels in another 84 lung cancer patients and then analyzed the differences of blood IGFBP2 among the lung cancer patients on the basis of histological diagnosis, clinical stage, and tumor size. The results showed that the mean IGFBP2 levels in male patients (382.38±248.81ng/ml) were significantly higher than those in female patients (303.58±209.73ng/ml, *P*=0.024). There was a trend for IGFBP2 to increase as the disease stage progressed from stage I to stage IV. The difference in IGFBP2 values between subgroups of patients with stage I and stage IV disease was significant (stage I: 259.42±187.38 ng/ml, stage IV: 383.6±226.36ng/ml; *P*=0.001; Table 1), suggesting that IGFBP2 is increased when the disease is progressed to advanced stage. The blood IGFBP2 value was mildly but also significantly correlated with the tumor size (R= 0.21, *P*=0.023) (Figure 2A). To determine whether blood IGFBP2 levels reflect IGFBP2 expressions in patients’ primary tumors, we analyzed IGFBP2 expressions in two primary tumor tissues from patients with either high or low IGFBP2 in their blood samples. Immunohistochemical analysis showed that the IGFBP2 expression was not detectable in the primary tumor of a patient with low blood IGFBP2 level but was markedly elevated in the tumor of a patient with high blood IGFBP2 (Figure 2B), suggesting that blood IGFBP2 levels may reflect IGFBP2 expression in the tumors. However, investigating on a larger set of patient samples with paired blood and tissue specimens will be required to determine the correlations between blood and tumor IGFBP2 levels.

Nevertheless, the difference between subgroups defined by tumor histology was not significant, although the mean value in cases of small-cell cancer was relatively higher than that for other histologies (small cell lung cancer: 389.7±222.28 ng/ml, non-small cell lung cancer: 310.69±143.09ng/ml, adenocarcinoma: 334.82±227.39ng/ml, squamous cell carcinoma: 315.47±251.71ng/ml, *P*=0.099). There was no significant difference in plasma IGFBP2 when patients were grouped by the median age of the patient population (age<52 years: 300.29±183.29 ng/ml, ≥52 years: 370.8±261.35ng/ml, *P*=0.12), or smoking status (never smoking: 306.33±215.95 ng/ml, ever smoking: 370.8±261.35ng/ml, *P*=0.34).

### Association of circulating IGFBP2 with clinical outcomes

**Figure 3:**
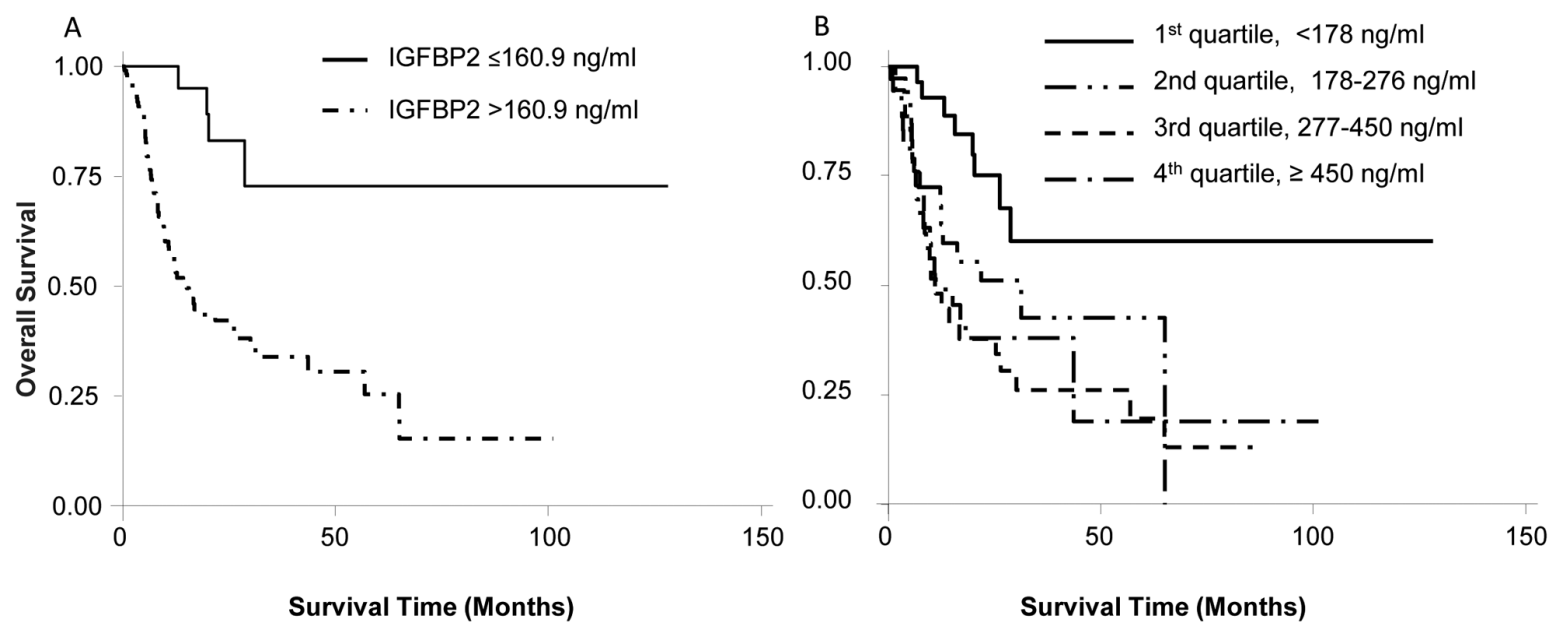
Association of blood IGFBP2 levels with survival time. A) The Kaplan-Meier survival curves for lung cancer patients grouped at the blood IGFBP2 cutoff value of 160.9ng/ml. The mean survival time for patients with higher IGFBP2 levels (N=120) was significantly shorter than that for patients with a lower IGFBP2 level (N=32, *P*=0.0002). B) The Kaplan-Meier survival curves for lung cancer patients subdivided into four groups on the basis of their blood IGFBP2 levels. Each quartitle has 38 patients. The mean survival time for the 1^st^, 2^nd^, 3^rd^, and 4^th^ quartiles were 128.16, 21.81, 11.11, and 10.92 months, respectively (*P*=0.033).

We then determined whether increased blood IGFBP2 was associated with patient survival. Univariate analysis showed that the survival is significantly correlated with histopathologies, disease stages, tumor sizes, and blood IGFBP2 levels (cutoff value at 160.9 ng/ml, which has the largest area under ROC curve) (Table 2). A multivariate analysis showed that lung cancer patients with blood IGFBP2 > 160.9 ng/ml had a poor survival outcome (hazard ratio = 8.76, 95% CI 1.12-68.34, p=0.038 after adjustment for tumor size, pathology, and stage) (Table 2). The median survival time for patients with IGFBP2 levels >160.9 ng/ml was significantly shorter than that for patients with a IGFBP2 level ≤160.9ng/ml (median survival time of 15.1 months vs 128.16 months, *P*=0.0002, Figure 3A). We also determined whether there was a survival difference when patients were divided into 4 groups on the basis of their blood IGFBP2 levels. The hazard ratio for poor survival increased as the IGFBP2 level increased from quartile 1 to quartile 4 (Figure 3B). Each quartile has 38 patients. The mean survival time for the 1^st^, 2^nd^, 3^rd^, and 4^th^ quartiles were 128.16, 21.81, 11.11, and 10.92 months, respectively (*P*=0.033). These results indicate that blood IGFBP2 levels could be a useful parameter for predicting patients’ outcomes.

**Table 2:** Univariate and multivariate analyses on survival hazard ratios.

**Variable**	**Alive, N(%**)	**Dead, N(%**)	**Hazard Ratio**	**95% CI**	**P value**
Sex					
Male	35 (52.24)	32 (47.76)	1		
Female	48 (56.47)	37 (43.53)	0.79	0.49-1.27	0.325
Age			1.00	0.98–1.03	0.944
Smoking status					
Never	14 (56.00)	11 (44.00)	1		
Former	19 (50.00)	19 (50.00)	1.05	0.50-2.23	0.891
Current	50 (56.18)	39 (43.82)	1.15	0.59-2.26	0.681
Tumor histology					
Adenocarcinoma	50 (60.02)	33 (39.75)	1		
Squamous-cell carcinoma	22 (68.75)	10 (31.25)	0.58	0.28-1.18	0.132
Non-small-cell carcinoma	7 (46.67)	8 (53.33)	1.09	0.50-2.37	0.835
Small-cell carcinoma	4 (18.18)	18 (81.82)	2.67	1.45-4.90	0.002
Disease Stage					
Stage I	29 (87.8)	4 (12.12)	1		
Stage II	10 (76.92)	3 (23.08)	3.07	0.62-15.26	0.169
Stage III	22 (56.41)	17 (63.59)	7.01	2.03-24.23	0.002
Stage IV	22 (32.84)	45 (67.16)	12.44	3.81-40.65	0.000
Tumor Size			1.19	1.05-1.35	0.005
Metastasis					
No	61 (66.30)	31 (33.70)	1		
Yes	22 (36.67)	38 (63.33)	1.56	0.88-2.77	0.130
IGFBP2					
≤160.9	29 (87.88)	4 (12.2)	1		
>160.9	54 (45.38)	65 (54.62)	5.58	2.03-15.34	0.001
**Multivariate** (IGFBP2)^*^					
≤160.9	29 (87.88)	4 (12.2)	1		
>160.9	54 (45.38)	65 (54.62)	8.76	1.12-68.34	0.038

^*^Adjusted by histology, stage and tumor size

## Discussion

Our results showed that blood IGFBP2 levels were significantly higher in lung cancer patients than in healthy controls, which are consistent with a recent study on serum IGFBP2 levels in 98 lung cancer patients, 17 benign lung disease and 23 normal controls in China, in which serum IGFBP2 levels and its autoantibodies were significantly elevated in lung cancer patients [33]. However, another recent study on 163 patients with different types of cancers, including 34 lung cancer patients, and 13 healthy controls in Germany showed that serum IGFBP2 levels were only significantly elevated in head and neck tumors but not in other types of cancer, including lung cancer [34]. It is not clear whether the discrepancy with the German study is due to the sample size or patient populations. Nevertheless, our study showed that the increase of blood IGFBP2 was associated with disease progression and poor outcome after adjusting for other clinical parameters, suggesting that the blood IGFPB2 level could be a useful parameter for predicting clinical outcomes.

Our study indicates that the blood IGFBP2 level alone might be not specific or sensitive enough for lung cancer diagnosis because both specificity and sensitivity were about 75%. It is not clear whether combining the IGFBP2 level with other biomarkers or screening approaches would increase its specificity and sensitivity. Indeed, although a number of blood biomarkers have been identified and evaluated in lung cancer patients, none has by itself shown the overall specificity and sensitivity sufficient for lung cancer diagnosis [35–37]. Because lung cancer is a heterogenic disease, it might be not realistic to expect any single molecule to be uniformly elevated in all lung cancer patients. We speculate that those markers might be used together to increase the specificity and sensitivity of screening tests or be used together with other screening methods to increase their specificity. For example, low-dose CT scanning has been found to be useful for early diagnosis of lung cancer, although the false positive rate is about 96% [2]. Blood markers may provide a noninvasive way to detect true positives in patients with suspected lung cancer on CT.

We found about 10% of healthy controls had a blood IGFBP2 level of 437ng/ml or higher. It is not clear whether those individuals were normal variants or had undiagnosed disease. A limitation of this work is that follow-up clinical information was not available to resolve this question. Because blood IGFPB2 was reported to be inversely associated with body mass in healthy people [38], we suspect that blood IGFBP2 could be high in some healthy people with very low body mass. Although plasma IGFBP2 concentrations in healthy human adults had minimal fluctuations in a 48-hour sampling period and did not change significantly post-prandially or after a glucose infusion, plasma IGFBP2 was increased about 1.7-fold in patients with extreme insulin deficiency or after 9 days of fasting [39].

Our recent proteomic study on primary NSCLC tumor tissues showed that a subset of lung cancer patients had a dramatic increase of IGFBP2 in their primary tumor tissue [22]. However, because the samples were from different sets of patients, it was not clear whether increased blood IGFBP2 was associated with increased IGFBP2 expression in tumor tissues, although such a correlation was observed in two paired samples. Nevertheless, consistent with the observation in primary tumor tissues, blood IGFBP2 was also increased in a subset of our study’s lung cancer patients. It will be of interest to investigate whether increased IGFBP2 in blood or primary tumor represents specific changes in signal transduction, metabolism, or biological functions in lung cancer, as was reported in glioma; in that cancer, increased IGFBP2 might serve as a biomarker for PTEN status and PI3K/AKT activation [40] or Ink4a-Arf status [41].

The association of increased blood IGFBP2 with shorter overall survival reported here in lung cancer patients and by others in ovarian cancer, colorectal cancer, and prostate cancer [28,29,31] suggests that IGFBP2 may contribute to malignant evolution and progression. Indeed, the IGFBP2/integrin/ILK/NF-kB network was recently reported to be a key player in glioma progression and poor outcomes [42]. Increased expression of IGFBP2 caused by loss of miR-126 was found to promote breast cancer cell metastasis by recruitment of endothelial cells to the cancer site through up-regulating the insulin growth factor 1 receptor (IGF1R) signaling pathway [12]. As a molecule capable of binding to IGFs, integrins, and extracellular matrix [6], IGFBP2 likely plays important roles in signal transduction and metabolic homeostasis in tumors, including exerting an autocrine effect on cancer cells and paracrine effects on endothelial cells and other mesenchymal cells. Characterization of IGFBP2-mediated pathways in lung cancer cells may provide molecular insights into lung cancer progression and potential therapeutic targets for lung cancer patients with increased IGFBP2.

## Materials and Methods

### Patient Samples

We matched blood samples from 80 lung cancer patients and 80 healthy controls for age, sex, race, and smoking history. We obtained these samples from the blood sample bank maintained in the Department of Epidemiology in our institution. Blood samples of another 84 lung cancer patients were either similarly obtained or collected from patients who had surgery at The University of Texas M D Anderson Cancer Center between 2007 and 2011. The blood samples were collected with either heparin or ethylenediaminetetraacetic acid (EDTA) as anticoagulation agents. Plasma was isolated by centrifuging the samples at 1500g for 5 min at room temperature. A pilot study on three healthy controls showed no significant difference in IGFBP2 levels in serum or plasmas obtained with either heparin or EDTA from the same person. All lung cancer patients and healthy volunteers had signed informed consent forms for sample collection. This study was approved by the ethics committee of The University of Texas M D Anderson Cancer Center. Plasma samples were frozen and maintained at -80°C until the assays were conducted.

### Enzyme-linked immunosorbent assay (ELISA)

Plasma IGFBP2 was measured by an enzyme-linked immunosorbent assay (ELISA) kit (Cell Sciences, Canton, MA, USA), following the manufacturer’s instructions. Briefly, plasma samples were diluted 1:200 with dilution buffer. One hundred microliters of diluted samples and serially diluted IGFBP2 standards were added in duplicate into each well of a 96-well plate pre-coated with IGFBP2 capture antibody. After incubating at 4°C overnight, the plates were washed with phosphate buffered saline containing 0.05% Tween 20 and incubated with biotinylated anti-human IGFBP2 detection antibody and horseradish peroxidase-conjugated streptavidin. The enzymatic activity of horseradish peroxidase was determined with its substrate 3,3’,5,5’-tetramethylbenzidine by measuring absorbance at 450 nm using a 96-well plate reader (Dynatech International, Chantilly, VA, USA). IGFBP2 concentration was calculated from the standard curve.

### Immunohistochemical Staining

Immunohistochemical staining was performed at the Histology Core Laboratory of our institution with Lab Vision Autostainer 360 (Thermo Scientific). Formalin-fixed and paraffin-embedded tissue sections (5-µm thick) were deparaffinized, hydrated, and heated in a steamer for antigen retrieval. The slides were then incubated with rabbit anti-human IGFBP2 antibody (Cell Signaling Technology). Rabbit anti-human IgG were used as a negative control for the primary antibody. The horseradish peroxidase conjugated secondary antibody was provided by the Histology Core Laboratory.

### Statistical analysis

We used statistical software Stata 10.1 for Windows (StataCorp LP, College Station, TX, USA) to carry out all statistical analyses. Values of IGFBP2 are expressed as mean ± standard deviation (SD). We analyzed demographic data with the chi-square test and compared continuous data with an independent-sample Student t-test. We compared the differences in plasma levels of IGFBP2 between cases and controls using the nonparametric Mann-Whitney U test and evaluated the relationship between IGFBP2 levels and clinical parameters by univariate linear regression analysis. We constructed survival curves according to the plasma levels of IGFBP2 using the Kaplan-Meier method and compared median survival times by the log-rank test. Hazard ratios and 95% confidence intervals for survival were estimated by multivariate Cox proportional hazard regression analysis, adjusting for tumor size, disease stage, and tumor histology. All *P* values were two-tailed with 0.05 specified as the threshold for statistical significance.
